# Epidemiologic profile of hemoglobinopathies in Benin

**DOI:** 10.1016/j.htct.2024.07.008

**Published:** 2024-10-08

**Authors:** Selma Gomez, Adjile Edjide Roukiyath Amoussa, Edwige Dedjinou, Manasse Kakpo, Pélagie Gbédji, Nouhoum Amossou Soulé, Bernice Quenum

**Affiliations:** a Centre de Prise en charge Médicale Intégrée du Nourrisson et de la Femme Enceinte atteints de Drépanocytose (CPMI-NFED), Benin; b Laboratory of Biology and Molecular Typing in Microbiology, University of Abomey-Calavi, Benin; c Laboratory of Cell Biology, Physiology and Immunology, Faculty of Sciences and Technology, University of Abomey-Calavi, Benin

**Keywords:** Sickle cell disease, Screening, Prevention, Mortality, Morbidity

## Abstract

**Background:**

Sickle cell disease is the most common inherited blood disorder in the world with the birth of approximately 300,000 newborns screened each year. In 2009, the World Health Organization ranked the fight against sickle cell disease among the priorities for the Africa regions. The best way to prevent this incurable disease remains, on one hand systematic screening at birth, and on the other the proscription of risky union between heterozygous subjects.

**Aim:**

The aim of this study was to analyze the epidemiological profile of sickle cell disease and other hemoglobinopathies in Benin and determine more up-to-date prevalence rates of the disease within the population.

**Methods:**

The hemoglobin profiles of 2910 study participants were determined by quantitative electrophoresis. Samples with abnormal hemoglobin results were subjected to a complete blood count.

**Results:**

Our study population was balanced between males (1528) and females (1382) with a sex ratio of 1.1. The mean age ranged from eight years in the pediatric group to 26 years in adults. The hemoglobin electrophoresis profiles found were as follows: 59.7 % Hb AA (normal), 21.7 % Hb AS, 10.2 % Hb AC, 3.1 % Hb SS, 3.7 % Hb SC, and 1.6 % of the rare phenotypes (Hb AD, Hb AE, Hb AF, Hb A/β-thal, Hb SD, Hb SF, Hb CC and Hb C/β-thal). Participants with abnormal hemoglobin presented a normochromic normocytic anemia. A total of 356 (12 %) people knew their profile compared to 2554 (88 %) who did not.

**Conclusion:**

The high prevalence of hemoglobinopathies found in this study highlights in importance of screening in the Benin population.

## Introduction

Hemoglobinopathies are inherited disorders that affect the structure or production of the hemoglobin (Hb) molecule.^1^ These are autosomal recessive disorders characterized by either reduced synthesis of Hb polypeptide chains in red blood cells (thalassemia) or structural changes in Hb (sickle cell disease - SCD).^2^

SCD, common to sub-Saharan Africa and regions of high black African migration, affects approximately 7 % of the world's population with approximately 300,000 to 400,000 sickle cell births occurring each year.^3^, ^4^, ^5^ It is often diagnosed by clinical manifestations of anemia, recurrent infections, and painful crisis affecting multiple organs.^6^^,^^7^ According to the World Health Organization (WHO) regional report for Africa, the burden of SCD in African regions is increasing together with the population growth creating serious consequences for public health and the socioeconomic situation of populations.^8^ In Benin, according to research, it is estimated that >4.8 % of the population is affected by major sickle cell syndromes; the prevalence of sickle cell traits is 22.3 % for Hb S compared to 10.21 % for Hb C.^9^, ^10^, ^11^ Thus, the high prevalence among Beninese populations makes SCD a real public health problem.

Because of the lack of a systematic neonatal screening program and national health surveillance of SCD, there are no data on the number of births affected by this pathology or the current prevalence of hemoglobinopathies in Benin. Therefore, with the aim of filling this paucity of information, data were obtained from the awareness and screening campaign activities carried out by the Centre de Prise en Charge Médicale Intégrée du Nourrisson et de la Femme Enceinte atteints de drépanocytose (CPMI-NFED - Newborn Screening for SCD and Comprehensive Clinical Care Program of Benin) in order to analyze the epidemiological profile of SCD and other hemoglobinopathies in Benin. CPMI-NFED is the national reference center, responsible to implementing strategies and directives to fight against SCD in Benin.

## Method

This is a selective or targeted study carried out during awareness campaigns (2021–2022) in selected universities, high schools and educational environments in Benin. The study was extended to hospitals, particularly to the pediatrics units of the National University Hospital Center - Hubert Koutoukou Maga (CNHU-HKM) in Cotonou, Zou-Colline Departmental Hospital Center in Abomey and Borgou-Alibori Departmental University Hospital Center in Parakou, during routine consultations or child vaccination appointments. The study was carried out in 13 cities and 13 municipalities in Benin were visited. These locations were selected based on the origin of children with SCD and previous studies.

Demographic information was collected for each participant, including age, sex, geographic origin, ethnicity, personal and/or family history and possible consanguinity. A blood sample was collected in an ethylenediaminetetraacetic acid (EDTA) tube by venipuncture for each subject in this study. Electrophoretic profile analysis was performed by Hb quantification in a high-pressure liquid chromatography (HPLC) instrument (Capillarys 2-Sebia, Variant™, Bio-Rad Laboratories, USA). A complete blood count was carried out to confirm homozygous or double heterozygous Hb abnormalities. Complete blood counts (CBCs) were carried out for all individuals who had homozygous or double heterozygous abnormalities using the Sysmex XT-4000i equipment. All clinical tests were performed in the clinical laboratory of CPMI-NFED.

In this study, all participants with major sickle cell syndromes (Hb SS, Hb SC, ß-thalassemia) or sickle cell profiles (e.g. Hb SD, Hb SF, Hb CC, Hb C/ß-thal, etc.…) were included in the hemoglobinopathies category.

### Statistical analysis

Statistical analyses were carried out using Excel and Epi-info software version 7.2. Quantitative variables were expressed as means and standard deviations and qualitative variables as numbers and percentages. The chi-square test was used for comparison tests with a p-value <0.05 being considered significant.

### Ethical considerations

The CPMI-NFED is a center for care, training and research. As such, it is able to carry out prospective studies under the guidance of an internal scientific advisory board. So, the principle and methodology of the awareness and screening campaigns carried out by the center in the Beninese population were validated by this scientific council. Prior to blood collection, the context of the screening campaign was fully explained to the participants followed by the signing of the free and informed consent form and the completion of data sheets. Approval was given by local authorities.

Confidentiality of patient data was maintained throughout the study. Data were kept anonymous by assigning codes to each participant. The study adhered to good clinical practice protocols and the ethical rules of the Declaration of Helsinki.

All participants received their signed and dated quantitative electrophoresis results. Participants with hemoglobinopathies were recalled and offered advice and guidance in a medical follow-up together with management of the disease.

## Results

A total of 2910 subjects participated in the study, including 1528 males (52.5 %) and 1382 females (47.5 %), with a sex ratio (M/F) of 1.1. The mean age of the entire cohort ranged from 8 ± 6 years in the pediatric group to 26 ± 7 years in adult group (Table 1).

**Table 1:** Distribution of the study population by age and gender.

**Variable**	**n**	**Mean / Frequency**
**Pediatric group (1–18 years)**		
Age	1012	7.927 ± 6.095
Gender		
Male	501	49.5 %
Female	511	50.5 %
**Adult group (19 years and over)**		
Age	1898	25.89 ± 6.98
Gender		
Male	1027	54.1 %
Female	871	45.9 %

Electrophoresis test results revealed the presence of common phenotypes (Hb AA, Hb AS, Hb AC, Hb SS, Hb SC) and rare phenotypes (Hb AD, Hb AE, Hb AF, Hb A/β-thal, Hb SD, Hb SF, Hb CC and Hb C/β-thal). The highest frequency was for normal Hb AA subjects (59.7 %), followed by Hb AS and Hb AC trait carriers with 21.7 % and 10.2 %, respectively. The prevalence of major sickle cell syndromes (Hb SS and Hb SC) was 6.8 %, that is, 3.1 % for the Hb SS profile and 3.7 % for the Hb SC profile. The prevalences of rare phenotypes was 1.6 %, that is, 0.8 % for the Hb AD, Hb AE, Hb AF, Hb A/β-thal and 0.8 % for the Hb SD, Hb SF, Hb CC and Hb C/β-thal profiles (Table 2).

**Table 2:** Distribution of electrophoretic profiles in the study population.

**Hemoglobin profiles**	**Male**	**Female**	**n**	**Frequency (%)**
**Normal**				
Hb AA	902	835	**1737**	**59.7**
**Trait carriers**				
Hb AS	344	288	**632**	**32.7**
Hb AC	166	131	**297**	
Hb AD	9	4	**13**	
Hb AE	1	0	**1**	
Hb AF	1	0	**1**	
Hb A/β-thal	4	3	**7**	
**Hemoglobinopathies**				
Hb SS	42	49	91	7.6
Hb SC	50	57	107	
Hb SD	1	3	4	
Hb SF	2	1	3	
Hb CC	5	11	16	
Hb C/β-thal	1	0	1	
TOTAL	1528	1382	2910	100

Of the 222 subjects diagnosed with hemoglobinopathies, approximately 28 % (*n* = 62) were in the 0–5 age group, 45 % (*n* = 100) were in the over 16 age group, and 16 % (*n* = 36) and 11 % (*n* = 24) were in the 6–10 and 11–15 age groups, respectively (Table 3).

**Table 3:** Distribution of hemoglobinopathies group by age range.

Profile	Age range (years)			
	1–5 years	6–10 years	11–15 years	>16 years
Hb SS	44	17	10	20
Hb SC	14	17	13	63
Hb SD/SF	0	0	0	7
Hb CC //β-thal	4	2	1	10
**Total**	**62**	**36**	**24**	**100**

Participants' prior knowledge of their electrophoretic profile was evaluated in this study. A total of 356 (12 %) people knew their profile compared to 2554 (88 %) who did not. Of the participants who had no prior knowledge of their phenotype, 63 % (*n* = 1608) are Hb AA, 34 % (*n* = 867) trait carriers and 3 % (*n* = 79) have hemoglobinopathies. However, the group of participants who had prior knowledge of their profile was dominated (40 %) by the hemoglobinopathy group (Hb SS, Hb SC and Hb CC), followed by normal Hb AA profiles (36 %) and trait carriers (24 % - Table 4).

**Table 4:** Previous knowledge of hemoglobin profiles.

**Pediatric group (*n*=1012)**			
**Hemoglobin profile**	**Yes(*n*=148)**	**No(*n*=864)**	**p-value**
**Normal**	21	485	<0.0001
**Trait carriers**	27	334	
**Hemoglobinopathy**			
Hb SS	65	13	
Hb SC	34	23	
Hb CC/ Hb C/β-thal	1	7	
Hb SD/ Hb SF	0	2	
**Adult group (*n*=1898)**			
**Phenotype**	**Yes(*n*=208)**	**No(*n*=1690)**	**p-value**
**Normal**	108	1123	<0.0001
**Trait carriers**	57	533	
**Hemoglobinopathy**			
Hb SS	11	02	<0.001
Hb SC	31	19	
Hb CC/ Hb C/β-thal	1	8	
Hb SD/ Hb SF	0	5	

An analysis of the CBC profile of subjects with hemoglobinopathies was performed and showed normochromic normocytic anemia in Hb SS subjects with Hb levels varying between 6 g/dL and 9 g/dL. Composite heterozygotes had mean Hb levels of approximately 13 g/dL for Hb SC and Hb SD subjects and approximately 11 g/dL in Hb SF and Hb CC subjects (Table 5).

**Table 5:** Hematological characteristics of subjects with hemoglobin abnormalities.

**Parameter**	**Hb SS**	**Hb SC**	**Hb SD**	**Hb SF**	**Hb CC**
Red Blood Cell count (x 10^12^/L)	2.7	5.08	6.0	3.71	4.8
Hemoglobin level (g/dL)	8.0	12.1	12.4	11.2	11.3
Mean Corpuscular Hemoglobin Concentration (pg)	38.5	23.4	30.9	36.6	32.3
Mean Corpuscular Volume (fL)	76.8	64.1	66.0	80.1	74.8
Mean Hemoglobin Concentration (%)	29.5	36.6	20.4	28.8	27.1
White Blood Cell count (g/L)	22.3	6.9	8.0	16.0	9.0
Platelet count (g/L)	385	241	2.0	261	357

## Discussion

This study, based on the identification of Hb alterations in the Beninese population, aims to reduce the ignorance of this population and thus the incidence and mortality due to SCD. The study is of interest because it took place in a country where there is no systematic screening for SCD and where the disease is usually diagnosed in the face of a serious complication.^8^ Studies conducted in Africa have shown that the probability of early death among children in sub-Saharan Africa is about 90 % in rural areas with limited access to health care, compared to 50 % in urban areas with better access and low exposure to infectious diseases.^11^ This clearly justifies the selection of the different sites included in this study.

A total of 2910 individuals were screened in this study with a mean age of eight years in the pediatric population and 26 years in the adult population. The population was gender balanced with a sex ratio (M/F) of 1.1. However, there was a predominance of females over males in the pediatric population, which is consistent with the standard age pyramid of the Beninese population.^12^

According to the results of the 2018 annual report of the National Institute of Statistics and Economic Analysis of Benin (INSTAD), the age of first sexual intercourse is estimated to be <15 years for girls and 17 years for boys. Moreover, the average age of first child birth is 17.8 years for girls and 19 years for boys, justifying the strategic choice of a predominantly young cohort (15–26 years) of this study.^13^ These figures highlight the precociousness of both sexuality and first childbearing among young adolescents, and therefore the urgency of implementing strategies to inform, sensitize and screen this population. This will make it possible, on the one hand, to reduce the number of couples at risk of giving birth to children with sickle cell anemia through knowledge of Hb electrophoresis and, on the other hand, to ensure early neonatal diagnosis of these children.

At the end of the screening, we found that the prevalence of normal subjects (Hb AA) was 59.7 %, while that of trait carriers was 32.7 % of the total study population, i.e. 21.7 % (Hb S) and 10.2 % (Hb C) for sickle cell traits. These results are similar to those of several other epidemiologic studies conducted in Africa, which estimate the prevalence of trait carriers in African regions to be from 10 to 40 %.^8^^,^^9^^,^^14^^,^^15^ Even if carriers do not show any symptoms of the disease, they are at risk of having children with the disease. They are therefore by far the group responsible for the increase in incidence in the global population over the years. Based on the 2018 annual report of INSTAD, which estimates the number of children per couple in Benin at 5.7, we could expect >3000 sickle cell births from these trait carriers. Information, education and communication (IEC) actions are therefore urgently needed to discourage the union of carriers of abnormal hemoglobins.

The prevalence of subjects screened for hemoglobinopathies was 7.6 % (*n* = 222), i.e. 6.8 % (Hb SS: 3.1 %; Hb SC: 3.7 %) for major sickle cell syndrome and 0.8 % for rare profiles. These results are much better than those obtained by several authors in Benin and other West African countries. Even if this difference can be attributed to the small cohort size of each of these studies compared to the current research, it is sufficient evidence that the prevalence of SCD in Benin is increasing with population growth.^8^^,^^9^^,^^13^ Furthermore, the prevalences of the Hb S and Hb C alleles were 16 % and 7.5 %, respectively, with associated incidences of 1:4 for the Hb S allele and 1:7 for the Hb C allele. In addition, the prevalence of the Hb CC profile was found to be 0.5 % in this study, which is higher than those found by Noudamadjo et al. and Zohoun et al. in Benin. Taken together, these results show a higher frequency of Hb S compared to Hb C in the study population, a fact consistent with the literature.^11^^,^^16^ Nevertheless, the results of the meta-analysis by Piel et al. justify the presence of the C allele due to the proximity of Benin to Burkina-Faso (the cradle of the C allele), Niger, Togo and Ghana.^17^, ^18^, ^19^, ^20^

The distribution of sickle cell profiles according to age at the time of this study (Table 3) shows a high frequency of Hb SS and Hb SC subjects in the 0–5 and over 16 age groups, respectively. The high frequency of Hb SS subjects in the 0–5 age group in the current study confirms the fact that the reasons for consultation are more obvious in Hb SS (homozygous) than in SC (heterozygotes) subjects, with the onset of the first complications in this period of the patient's life.^21^^,^^22^ In developing countries such as Benin, diagnosis is rarely made before the age of two years due to the lack of systematic neonatal screening.^23^ This underscores the importance of implementing a systematic sickle cell screening strategy in maternity clinics to significantly reduce late diagnosis and associated infant mortality. On the other hand, the high frequency of heterozygous profiles (Hb SC, Hb SD, Hb CC and Hb C/β-thal) detected in over 16-year-old subjects could be explained by the fact that these are almost asymptomatic forms of SCD, i.e. less expressive in terms of severity of manifestations. Although SC heterozygosity is an integral part of the major sickle cell syndromes, it sometimes presents with less severe symptoms than SS homozygosity. As a result, the subtle hematologic abnormalities and staturo-ponderal growth of SC heterozygotes, which are nearly identical to those of the general population, often lead to a delay in diagnosis into adulthood.^24^^,^^25^ However, the natural history of SC heterozygotes, characterized by the onset, of often irreversible complications, due to hyperviscosity and hypercoagulability of the blood, also requires early diagnosis, as in SS homozygotes. According to the literature, the rare hemoglobinopathy profiles identified in the current study are mostly asymptomatic, but may be responsible for the increased incidence of SCD in the general population if diagnosis is delayed or misdiagnosed.^8^^,^^26^

The assessment of prior knowledge of the disease in this study is related to information on the electrophoretic status available to patients. Thus, approximately 88 % of the study population (864 pediatric; 1690 adult) were unaware of their electrophoretic profile, compared to 12 % (148 pediatric; 208 adult) who had some idea. Of those unaware of their profile, 13 Hb SS and 23 Hb SC major sickle cell syndrome subjects were identified in the pediatric group and 2 Hb SS and 19 Hb SC individuals in the adult group. These data show that although SCD is a real health problem with a high mortality rate in African regions, it is still very little known among the Beninese population.^8^^,^^27^ The lack of systematic screening and awareness of SCD at all levels of the country's health pyramid could explain the high number of major sickle cell syndrome cases detected late in the pediatric unit of the CNHU-HKM in Cotonou.^10^ Comparing these data with the total number of hemoglobinopathies screened in this study, we can see that 64 % of subjects with severe SCD knew their status (with a predominance of Hb SS profiles), compared to 26 % who did not (with a predominance of Hb SC and uncommon profiles). This confirms that subjects with severe forms of SCD are rarely unaware of their status due to the often early onset of painful attacks and high susceptibility to infection. It is a fact that referral of patients to confirmatory screening by health care workers is a reality, given the obvious symptoms presented by these patients.

Biologically, this study found normochromic normocytic anemia in almost all Hb SS subjects and microcytosis in Hb SC, Hb SD, and Hb C/β-thal individuals. Almost all Hb SF and Hb CC subjects had normal blood counts. The anemia observed in the majority of subjects and the microcytosis characteristic of composite forms have been frequently reported in the literature.^11^^,^^28^^,^^29^ This test could be a primary indicator to identify patients with Hb abnormalities in rural areas where diagnostic facilities are almost non-existent.

## Conclusion

The results of the study show the different epidemiologic profiles of hemoglobinopathies in Benin. The presence of almost all of the different Hb types (Hb S, Hb C, Hb D, Hb F, β-thalassemia, etc.) in this study population shows that hemoglobinopathies, due to human migration, intercultural mixing and interracial marriages, are no longer limited to a specific geographical area.

Although these initial results are limited to 2910 individuals, they provide significant information on the prevalence of SCD for the Beninese health system. The high prevalence rates of major sickle cell syndromes show that greater efforts are still needed to better inform the population to adhere to systematic neonatal screening programs for this pathology.

Although screening and early medical management are essential to reduce mortality and the various disabilities caused by the disease, targeted screening remains the best means to reduce the number of couples at risk in African countries where the population is under-informed about the serious consequences and medico-social burden of the disease on patients, their families and society.

**Author Contribution:** SG, AERA and QB designed the study. SG, AERA, ED and QB supervised the study. PG, NAS, SG and MK performed the experiments and recorded the data. SG and AERA analyzed the data and wrote the manuscript. All authors have approved the final article.

## Funding

This work was supported by the 10.13039/501100006506Ministry of Health of Benin and the World Health Organization (WHO).

## Conflicts of interest

I confirm that there is no conflict of interest.
